# Study on the Influence of Accelerated Aging on the Properties of an RTV Anti-Pollution Flashover Coating

**DOI:** 10.3390/polym15030751

**Published:** 2023-02-01

**Authors:** Junwei Chen, Bo Li, Xiaomei Zeng, Zhenggang Li, Yi Wen, Quan Hu, Qiu Yang, Mi Zhou, Bing Yang

**Affiliations:** 1 Electric Power Research Institute of Guizhou Power Grid Co., Ltd., Guiyang 550002, China; 2 School of Power and Machinery, Wuhan University, Wuhan 430072, China; 3 Tongren Power Supply Bureau of Guizhou Power Grid Co., Ltd., Tongren 554300, China; 4 School of Electrical Engineering and Automation, Wuhan University, Wuhan 430072, China

**Keywords:** RTV, color difference, gloss, surface morphology, infrared reflection spectrum, static contact angle

## Abstract

The purpose of this work is to study the accelerated aging behavior of a room-temperature vulcanized (RTV) silicone rubber anti-pollution flashover coating. Red, blue and gray RTV rubber samples were selected to prepare coatings on the surface of stainless-steel sheets. The accelerated aging test was carried out in an aging test chamber according to a four-step program cycle. After the completion of different aging tests, the color difference, glossiness, surface micromorphology, wettability, insulation performance and other parameters of the samples were measured using colorimetry, infrared spectrometry, scanning electron microscopy, and a high-voltage breakdown tester. The results showed that with the increase in aging time, the color difference ∆*E_ab_* of the coatings increased. The G60 gloss value decreased gradually and tended to be saturated after 60 cycles. After the aging tests, the RTV coating surface had holes, cracks, peeling and other damage to varying degrees. The C:Si atomic ratio was less than 2, and the hydrophobicity was obviously deteriorated. After aging, the electrical strength of the three RTV coatings decreased significantly. It can be concluded that during the accelerated aging test, the RTV coating had cross-linking and oxidation reactions, and the internal deterioration and surface damage of the coating led to changes in its color, luster, morphology, insulation strength, etc.

## 1. Introduction

Insulator flashover caused by pollution flashover of transmission and distribution network lines is the main cause of power grid accidents, seriously affecting the safety and reliable operation of the power grid. The main reason for pollution flashover is that the solid or liquid pollution particles deposited on the surface of the line insulator cause flashover due to the reduction in the electrical strength of the insulator under certain weather conditions. The room-temperature vulcanized (RTV) silicone rubber coating is a very widely used anti-pollution flashover material [1]. The main component of RTV coating is polydimethylsiloxane (PDMS), which is a new type of anti-pollution and hydrophobic coating [2,3,4,5,6]. Externally insulated power equipment coated with an RTV coating does not require extensive cleaning and maintenance, and RTV coatings have thus been widely used. As an organic material, an RTV coating is ages quickly when exposed to severe environmental conditions such as heavy pollution, ultraviolet radiation, acid rain, high temperature and humidity [7], resulting in filler particles, holes and cracks, and molecular structure damage [8], which gradually reduces its hydrophobicity and hydrophobicity mobility, and has a great impact on the anti-pollution flashover performance of RTV coatings. Therefore, it is of great significance to accurately evaluate and detect the aging degree of RTV coatings.

A large number of studies show that temperature, humidity, ultraviolet radiation and other environmental parameters are the main factors affecting the aging speed of the RTV coatings. The aging speed in coastal or subtropical areas is much higher than that in dry areas [9,10,11]. The investigation by Can Chen et al. [12] showed that the high temperature and humidity over the course of the year in Guangdong were very likely to cause the RTV coating to pulverize, fade and peel off. Morphological changes on the RTV coating’s surface were observed by SEM, and the change in surface element composition before and after RTV coating aging was analyzed by means of the EDS/EDX technique [13]. D. Devendranath [14] and R.S. Gorur [15] used this element analysis method to compare the content and proportion of Si and Al in the RTV coating’s surface and a composite insulator before and after aging, respectively. The results show that the proportion of Si/Al decreases significantly with the aging degree of the sample, which means that the components in the RTV coating are damaged. Hao Yang et al. [16] established an RTV coating aging classification method based on surface roughness (surface particles, holes and cracks), the average particle size and element content by analyzing the micro morphology of RTV coating samples. However, the evaluation process is complex, which is not conducive to field application.

The anti-pollution flashover performance of the RTV material is determined by its surface state. Researchers have also carried out a lot of work in polymer surface state monitoring based on laboratory samples [17,18,19]. Based on X-ray diffraction analysis, B. Jia proposed that SiO_2_ composition can be used to evaluate the surface state of RTV [20]. Y. Xia established the relationship between thermal weight loss and RTV surface state [21]. Alok studied the performance change of silicone rubber insulators under various stress aging scenarios and reported the loss of aluminum trihydrate (ATH) filler in silicone rubber after aging [22]. In addition, Kumagai proposed a simple method to evaluate the state of the polymer surface by using the leakage current characteristics [23]. Rahmat analyzed hydrophobicity and performed mechanical tests, leakage current tests, FTIR and scanning electron microscope tests to study the effect of multiple stress aging [24,25]. Ullah I. et al. [26] studied the weatherability of RTV silicone rubber under high pressure, ultraviolet radiation and temperature stress. All sample surfaces showed obvious discoloration, which may be caused by the cyclization behavior in the polymer during the process of ultraviolet radiation, heat and high electrical stress.

To sum up, although the test results in an ambient atmosphere are relatively reliable, the aging speed is slow, the climate is unpredictable, and the controllability is poor, which is not suitable for basic scientific research and industrial production. Traditional artificial aging, which is based on an AC/DC multi stress accelerating chamber, high temperature annealing, a UV box, etc., can only simulate one situation in the complex real service environment. An aging test chamber with a xenon arc lamp can simulate the full sunlight spectrum to reproduce destructive light radiation in different environments, which is a widely used assessment method in the aging research of the polymer materials. In this study, xenon lamp weathering equipment is used for accelerated aging. The aging chamber with a xenon lamp with an optical filter can effectively simulate the solar spectrum at the Earth’s surface, and also can set up a rain environment, with high air temperature and high humidity.

## 2. Materials and Methods

### 2.1. Sample Preparation

The red, blue, and gray RTV rubber samples were purchased from Hebei Zhonglian Huayu Power Technology Co., Ltd., China. The RTV rubber consisted of polydimethylsiloxane (PDMS, CAS#63148-62-9), methyltriacetoxysilane (CAS#4253-34-3), aluminum trihydrate and aluminium hydroxide to improve arc resistivity, SiO_2_ reinforcing filler, and pigments.

The red, blue, and gray RTV rubbers were brush-coated onto 3 × 4 × 0.1 cm^3^ stainless steel sheets. The RTV coating of each color was created by performing the brushing three times, and the interval between two brushings was 24 h. The total thickness of the coatings was 0.3 mm. The samples were placed in an aging chamber for the accelerated aging test. The samples were taken out regularly to measure the color difference, glossiness, morphology, infrared spectra, static contact angle and other parameters.

Before the aging test, the element composition of the coatings was studied uisng the energy dispersive X-ray spectrometry (EDS), as shown in Table 1. The blue coating contained C, O, Al and Si; in the red coating, Fe_2_O_3_ was added; and the gray coating contained TiO_2_. The C:Si ratio was close to 2.

### 2.2. Accelerated Aging Test

**Figure 1:**
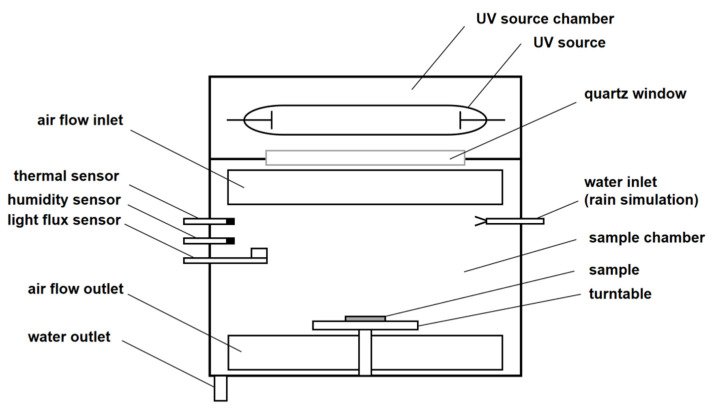
Schematic diagram of the test chamber.

A BT-SD-150A xenon lamp weathering test chamber (Shenzhen Beite Instrument Equipment Co., Ltd., Shenzhen, China) was used to conduct the accelerated aging test for RTV coatings. The schematic diagram of the test chamber is shown in Figure 1. The internal structure of the test chamber was composed of the sample chamber and the xenon lamp light source chamber, including an air-cooled xenon arc light source, a quartz glass filter, an automatic water spraying control device, sensors (temperature, humidity, luminous flux), a rotatable sample table, etc. The xenon arc lamp could simulate the full sunlight spectrum to reproduce the destructive light radiation. At the same time, the quartz glass filter was used to filter the xenon lamp radiation, so that the spectral energy distribution received by the sample surface was close to the natural solar spectrum, between 295 and 800 nm. The temperature and humidity in the sample chamber were controlled by air, with an appropriate temperature and humidity entering the chamber. Considering that the samples would be used in a high-humidity and solar irradiation environment, the four-segment light–dark cycle program mode was adopted to simulate dark, light, rain, sunlight and other cyclic environments. One cycle lasted for 3 h—see Table 2 for specific test parameters.

### 2.3. Experimental Setups

#### 2.3.1. Measurement of Color and Gloss

A BYK 4430 micro multi-angle glossmeter was used to measure the color difference and glossiness of the samples before and after aging. The glossiness was measured at the incidence angle of 60°. The arithmetic means of the five measurements were calculated. In this paper, the CIE LAB Color model was used to represent the change in color of the RTV coatings. The CIE LAB Color space is composed of L*, a*, and b* color space coordinates. L* represents the lightness, which is equivalent to the brightness, and a* and b* represent the range from magenta to green and from yellow to blue, respectively. The value range of L* is from 0 to 100. When *L* = 50, it is equivalent to 50% black. The range of a* and b* values is from −127 to +128. The total color difference of two measurements before and after aging can be calculated as an Euclidean distance in the color space using formula: $∆E_{ab}=\sqrt{{\left(L_{2}^{*}-L_{1}^{*}\right)}^{2}+{\left(a_{2}^{*}-a_{1}^{*}\right)}^{2}+{\left(b_{2}^{*}-b_{1}^{*}\right)}^{2}}$.

#### 2.3.2. Observation of Micro Morphology of Coating Surface

A scanning electron microscope (SEM, Tescan MIRA3, Brno, Czech Republic) operated at 20 kV was used to observe the change in the surface microstructure of the samples, and an Oxford Instrument X-MAX^N^ energy dispersive X-ray spectrometer (EDS) was used to detect the element distribution. The RTV coating is a good insulating and protective material; therefore, before the SEM/EDS tests, the surface of the samples was coated with a gold layer to increase its conductivity.

#### 2.3.3. Infrared Spectroscopy

The reflectance spectra in the near infrared region of the as-prepared and aged coatings were obtained using an INVENIO-S Fourier infrared spectrometer (Bruker, Heidelberg, Germany) to investigate the changes in the infrared characteristics of the functional groups. The number of scanning runs was 32, the resolution was 4 cm^−1^, and the scanning range was 400–4000 cm^−1^.

#### 2.3.4. Static Contact Angle Test

The static contact angle is a parameter that directly measures the hydrophobicity and surface failure of the coating. In this study, a contact angle measuring instrument (Shanghai Yinnuo Precision Instrument Co., Ltd., Shanghai, China) was used to measure the static contact angle of the RTV coatings before and after aging. The volume of deionized water drops used was 3 μL. The contact angle was measured at five points of each sample and the mean value was calculated.

#### 2.3.5. High Voltage Breakdown Test

The voltage breakdown test was carried out to test the insulation performance of the RTV coatings. The high-voltage measuring system FRC-200 kV AC/DC with a light high-voltage experimental transformer and other equipment provided by Wuhan Huadian Meilun Power Technology Co., Ltd., Wuhan, China, were used for voltage breakdown measurement. The highest value of the voltage before the coating failure was recorded. Both coatings before and after aging were tested. Five points of each sample were tested to obtain the mean value of the breakdown voltage.

## 3. Experimental Results and Discussion

### 3.1. Color and Gloss Change

**Figure 2:**
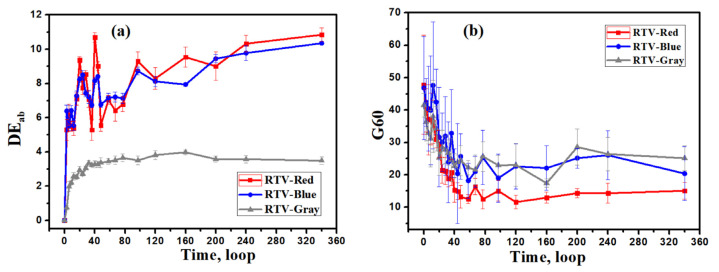
Changes in color difference (**a**) and glossiness (**b**) of coatings before and after accelerated aging.

Figure 2 shows the changes in color difference (a) and glossiness (b) of the three RTV coatings. The change trend of color difference and glossiness is consistent. The color difference gradually increases with the aging time. It increases greatly at the beginning, and then gradually slows down. Among the coatings, the color change of the red and blue coatings is more rapid than that for the gray coating. After 40 cycles, the color difference of the red and blue coatings reaches about 8, and that of the gray coating is about 3. For the glossiness, the overall glossiness shows a reducing trend which changes from fast to slow. The red coating loses its glossiness more rapidly than the blue and gray coatings. In 0 to 60 cycles, the red coating’s glossiness decreases from 48 to 12, and then stabilizes at around 12. The light retention rate is only 25%. The gray coating’s glossiness decreases from 41 to 17, and the light retention rate is 41%, while the blue coating’s glossiness decreases from 46 to 22, and its light retention rate is the highest among the three, reaching 48%. After 60 cycles, the degradation of the RTV coatings’ glossiness reaches saturation.

The color change of the gray coating is smaller than that of the red and blue ones, which may be due to a slight difference in composition, as shown in Table 1. EDS analysis reveals that C, O, Al and Si exist in the three colors of coatings, while a small amount of Fe and Ti are observed in the red and gray coatings, respectively, which were added to the coatings as Fe_2_O_3_ and TiO_2_ additives. However, the content of Fe and Ti elements is low, at only about 1%. At the same time, even though there are additional Fe and Ti elements in the red and gray coatings, the color difference change trend of the blue coating is comparable, indicating that the addition of Fe and Ti elements is unlikely to change the color of the coating after the aging test. Therefore, we explain the color change by the formation of products during the aging of PDMS and the curing agent. If the color of the aging products is different from the color of the initial material, we can observe the change in the total color.

### 3.2. Change in the Micro-Morphology and Element Composition

**Figure 3:**
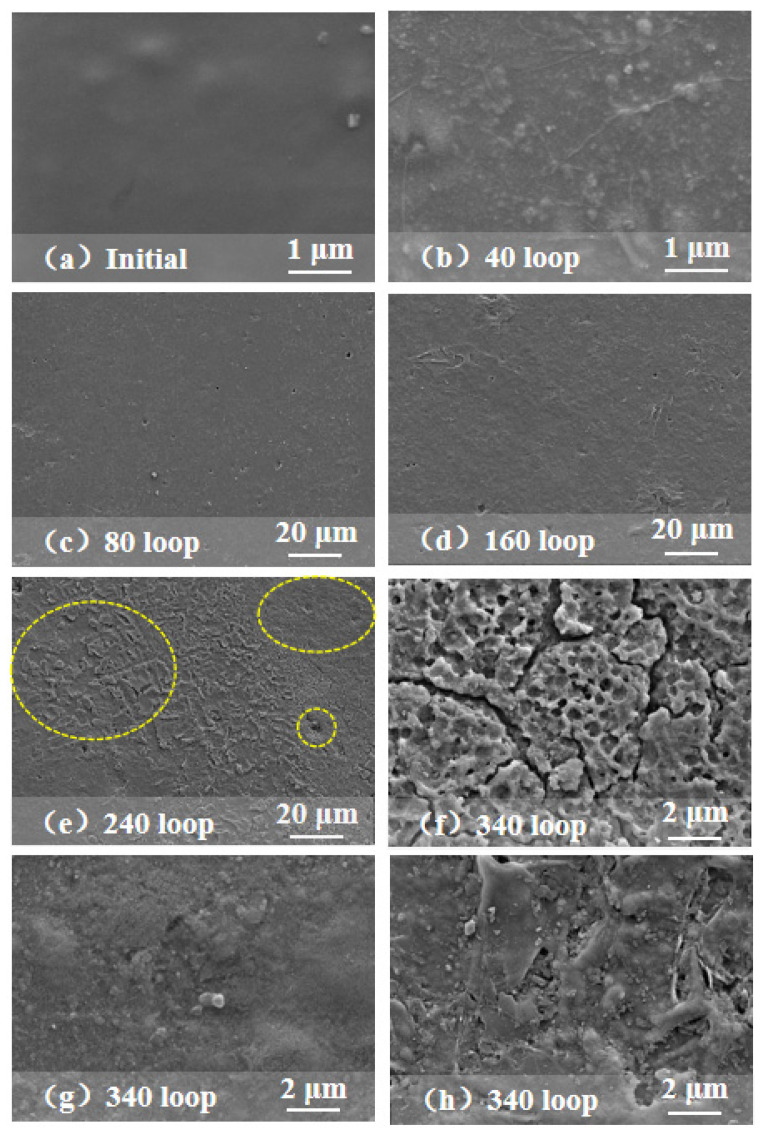
SEM surface morphology of RTV coatings after aging for different periods: (**a**–**f**) red coating, (**g**) blue coating, and (**h**) gray coating.

Figure 3 shows the SEM surface morphology of the red, blue and gray coatings after aging for different periods. The morphological damage of the blue coating is at a relatively lower level than that of the red and gray coatings, mainly showing holes. The gray coating has bubbles and peeling, while the red coating has the most serious structural damage, with multiple corrosion phenomena such as folds, holes, cracks, peeling, etc., and the peeling area presents a honeycomb surface.

It can be seen from Figure 3 that the surface of the red coating is relatively smooth and clean before aging. With continuous aging, the surface gradually becomes uneven, indicating that the microstructure of the sample is damaged to a certain extent. After the 40th cycle, there are small holes, undulations and wrinkles on the coating surface. This is due to the fact that during the aging process, the spectral wavelength is 295–800 nm, which promotes the cross-linking and oxidation reactions of methyl groups. Both reactions consume methyl and generate methane and formaldehyde, respectively:

Cross-linking reaction: $\left(-{\mathrm{CH}}_{3}\right)+\left(-{\mathrm{CH}}_{3}\right)\to \left(-{\mathrm{CH}}_{2}-\right)+{\mathrm{CH}}_{4}↑$

Oxidation reaction: $\left(-{\mathrm{CH}}_{3}\right)+O_{2}\to -\mathrm{OH}+\mathrm{HCHO}↑$

During the reaction process, CH_4_ and HCHO gases are generated and damage the originally flat surface. In the early stage of aging, it is mainly manifested as wrinkles [27]. After 160 cycles, the volume of CH_4_ and HCHO gases increases, and the coating presents with local cracks and peeling, while after 240 cycles, cracks and peeling spread over the entire surface. At the same time, because the cycle process also involves temperature alternation, stress fatigue accelerates physical aging, and the thermal expansion and cold contraction processes lead to the fracture of the internal cross-linked bonds and macromolecular chains of silicone rubber. When the silicone rubber deteriorates to a certain extent, the crack path gradually evolves into cracks [28,29]. The research shows that for polymer matrix composites, oxygen and heat promote the continuous initiation and development of coating cracks, which further act on the matrix through the vertical cracks in the coating to enhance oxidation [30]. Therefore, under the continuous effect of high temperature, high humidity and UV irradiation, after 340 cycles, the coating cracks further increase, and the surface even appears powdery, presenting a honeycomb surface, as shown in Figure 3f. Therefore, after the change in the color and gloss, when the coating reaches saturation, it does not mean that the aging degree is saturated, and its micro morphology is still deteriorating.

**Figure 4:**
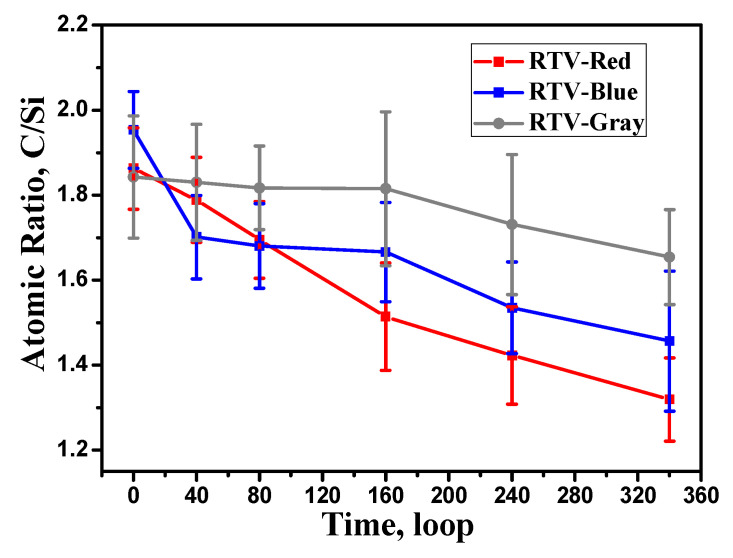
Average C:Si ratio of the RTV samples. EDS scanning area is 40 × 50 μm.

According to the molecular structure of PDMS, the relative ratio of C:Si is close to or greater than 2, which is consistent with the EDS results. The average C:Si ratio in samples at different aging times is shown in Figure 4. With the increase in aging time, the C:Si ratio of the samples shows a downward trend. The red RTV coating shows the fastest decrease in the C:Si ratio, while that of the gray RTV coating decreases the slowest. The C:Si ratio of the red, blue and gray coatings decreases from 1.9 to 1.3, from 2.0 to 1.5, and from 1.8 to 1.7, respectively. All C:Si ratios are less than 2, indicating that the molecular structure of the RTV surface has been seriously damaged. In the red coating, the C:Si ratio decreases the most, which is consistent with the highest surface damage degree observed by SEM, as shown in Figure 3. The reason for the decrease in the C:Si ratio is that the above cross-linking and oxidation reactions consume part of the CH_3_, and release CH_4_ and HCHO gases, resulting in carbon loss. Combined with the SEM surface morphology analysis, when the C:Si ratio of the coating is lower than 1.5, it means that the main PDMS component and the coating’s morphology have been seriously damaged.

### 3.3. Changes in IR Reflectance Spectra

**Figure 5:**
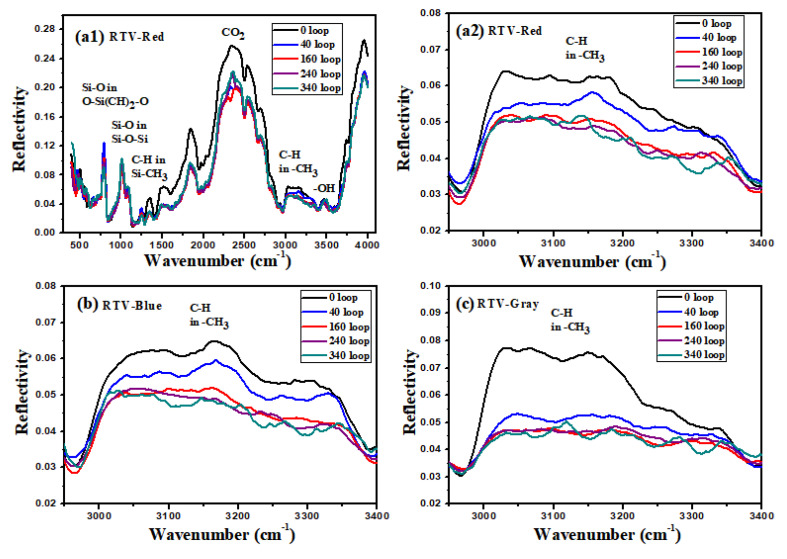
IR reflectance spectra of RTV coatings before and after aging: ((**a1**,**a2**)) red coating, (**b**) blue coating, and (**c**) gray coating.

FTIR can analyze the chemical bonds and groups of materials according to the peaks and areas in the reflection spectrum. The main functional groups in the RTV coating are shown in Table 3. It can be seen from Figure 5 that the reflectance near the characteristic wave numbers corresponding to the functional groups changes after aging. The changes in peak intensity of Si-CH_3_ near 1250 cm^−1^, C-H in -CH_3_ near 3000 cm^−1^ and O-H near 3500 cm^−1^ of the three coatings are consistent. After aging, because the cross-linking reaction and oxidation reaction [26] described above in Section 2.2 consume part of the CH_3_, the content of CH_3_ is significantly reduced. The CH_3_ group is a hydrophobic group, which means that the hydrophobicity of the coating will be weakened after aging. This result is consistent with the decrease in the static contact angle—see below. However, the content of the OH group hardly changes, because the oxidation reaction produces the OH on the surface, and at the same time, Al(OH)_3_ and other additives are decomposed, reducing the content of OH. Therefore, the rise and fall in OH content reflected in the IR are actually the comprehensive result of the oxidation and decomposition reactions of additives.

### 3.4. Change in the Static Contact Angle

**Figure 6:**
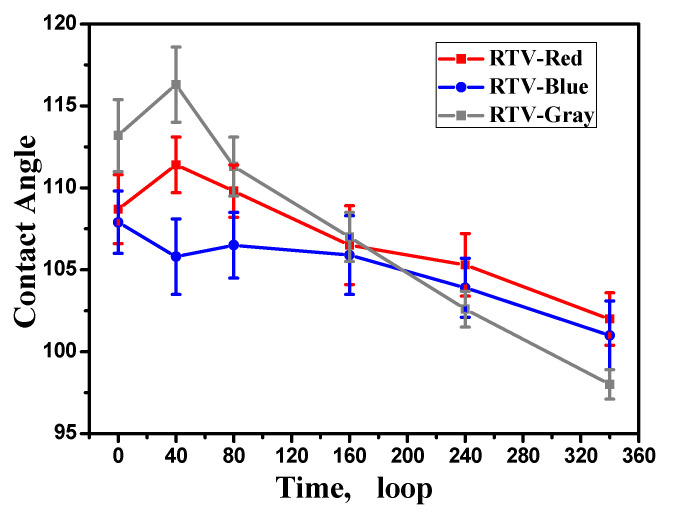
Static contact angle of RTV coatings before and after aging.

It can be seen from Figure 6 that with the increase in aging time, the static contact angle of the RTV coatings first slightly increases and then decreases gradually, which means that the hydrophobicity is weakened as a whole, which is consistent with the decrease in the content of hydrophobic CH_3_ radicals in the IR reflectance spectra. The initial static contact angles of the RTV coatings are higher than 108°. After 340 aging cycles, the contact angle of the red, blue, and gray coatings is reduced to 102°, 101° and 98°, respectively, but still meets the requirements of anti-pollution flashover. During the aging, the initial cross-linking reaction plays a leading role, and the UV radiation promotes the RTV cross-linking, so that it can be completely vulcanized; therefore, the static contact angle increases. Subsequently, due to oxidation and decomposition reactions, the hydrophobic CH_3_ groups are consumed and the hydrophilic OH groups are formed at the same time, the hydrophobicity of the coating is weakened, and the static contact angle is reduced. It is worth noting that after 340 cycles, the static contact angle of the red coating is the highest, and its hydrophobicity is better than that of the blue and gray coatings. This is related to the surface roughness of the coating—as shown in the SEM image, the red coating has the most serious morphological damage and the highest roughness.

### 3.5. High Voltage Breakdown Test

**Figure 7:**
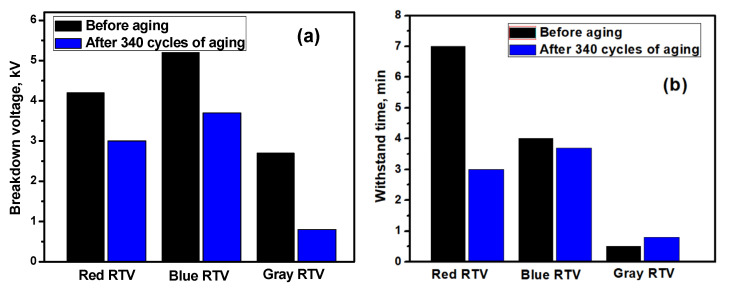
Breakdown voltage (**a**) and withstand time (**b**) of the RTV coatings before and after aging.

The breakdown voltage and withstand time before the aging and after 340 cycles were measured, and the results are shown in Figure 7. It can be seen from Figure 7 that the breakdown voltage and withstand time of the three coatings are significantly reduced after aging, which means that the insulation performance degrades, which is related to the damage to the molecular structure and surface morphology after aging. At the same time, it is worth noting that the breakdown voltage and withstand time of the gray coating are significantly lower than those of the red and blue coatings, which is due to the smaller thickness of the gray coating, which is only half that of the other coatings.

## 4. Conclusions

Through a systematic study on the effects of aging on the structure and morphology of the red, blue and gray RTV coatings, the following conclusions are obtained:

(1) With the increase in the aging time, the color difference of the three RTV coatings increases rapidly and then becomes stable, and the color difference of the gray coating is lower than that of the red and blue ones. The change trend of glossiness is opposite to that of the color difference. The glossiness of the red coating weakens faster than that of the blue and gray ones, and the light retention is 25%, 48% and 41%, respectively.

(2) During the aging of the coatings, because the cross-linking and oxidation reactions both consume methyl, the C:Si ratio decreases, CH_4_ and HCHO gases are produced, and the flat surface is damaged when the gases escape. Bubbles and wrinkles appear successively in the coating, and finally large cracks and peeling occur. Since the main components of the three coatings are the PDMS, they all contain common chemical bonds of silicone rubber. After aging, the change trend of each functional group is also consistent. The cross-linking and oxidation reactions consume part of the hydrophobic CH_3_ groups, while the reflection peak intensity corresponding to the hydrophilic OH groups changes little.

(3) With the increase in the aging time, oxidation and decomposition reactions play a leading role, the static contact angle is generally reduces, and the hydrophobicity is weakened. When the color difference and glossiness are stable, the coating micro surface, element ratio, functional group content and static contact angle still change according to a certain trend.

(4) After aging, the molecular structure and surface morphology of the RTV coating are seriously damaged, and so the breakdown voltage and withstand time are significantly reduced, and the insulation performance is degraded.

Gloss, color and voltage breakdown tests can be used to detect and evaluate the aging damage of the RTV coating.

## Figures and Tables

**Table 1:** Element composition of the RTV coatings.

	Atomic Percentage, %		
Element	Blue RTV	Red RTV	Gray RTV
C	30.1	31.5	36.8
O	43.4	41.3	34.8
Al	11.1	9.5	6.9
Si	15.4	16.9	20
Fe	-	0.8	-
Ti	-	-	1.5

**Table 2:** Four-segment light–dark cycle program for the accelerated aging test.

	1st Step	2nd Step	3rd Step	4th Step
Test temperature (°C)	38	47	47	47
Humidity (%)	95	50	95	50
Blackboard temperature (°C)	38	70	70	70
Irradiation intensity (W/m^2^)	0	800	800	800
Water spray	ON	OFF	ON	OFF
Time (min)	60	40	20	60

**Table 3:** The main functional groups in RTV coatings [31].

Functional Group	Wave Number/cm^−1^
Si(CH_3_)_3_	700
Si-O in O-Si(CH_3_)_2_-O	790~840
Si-O in Si-O-Si	1000~1100
C-H in Si-CH_3_	1255~1270
C-H	1410~1440
C-H in CH_3_	2960~2962
OH	3200~3700

## Data Availability

Not applicable.
